# A thorough annotation of the krill transcriptome offers new insights for the study of physiological processes

**DOI:** 10.1038/s41598-022-15320-5

**Published:** 2022-07-06

**Authors:** Ilenia Urso, Alberto Biscontin, Davide Corso, Cristiano Bertolucci, Chiara Romualdi, Cristiano De Pittà, Bettina Meyer, Gabriele Sales

**Affiliations:** 1 Department of Biology, University of Padova, Via U. Bassi 58/B, 35131 Padova, PD Italy; 2 Department of Life Sciences and Biotechnology, University of Ferrara, Via Luigi Borsari 46, 44121 Ferrara, FE Italy; 3 Biology and Evolution of Marine Organisms, Stazione Zoologica Anton Dohrn Napoli, Villa Comunale, 80121 Naples, Italy; 4 Alfred Wegener Institute Helmholtz Centre for Polar and Marine Research, Am Handelshafen 12, 27570 Bremerhaven, Germany; 5 Institute for Chemistry and Biology of the Marine Environment (ICBM), Carl-von-Ossietzky University, Carl-von-Ossietzky-Straße 9-11, 26111 Oldenburg, Germany; 6 Helmholtz Institute for Functional Marine Biodiversity (HIFMB) at the University of Oldenburg, 26111 Oldenburg, Germany

**Keywords:** Bioinformatics, Sequencing, Molecular biology, Ecology

## Abstract

The krill species *Euphausia superba* plays a critical role in the food chain of the Antarctic ecosystem. Significant changes in climate conditions observed in the Antarctic Peninsula region in the last decades have already altered the distribution of krill and its reproductive dynamics. A deeper understanding of the adaptation capabilities of this species is urgently needed. The availability of a large body of RNA-seq assays allowed us to extend the current knowledge of the krill transcriptome. Our study covered the entire developmental process providing information of central relevance for ecological studies. Here we identified a series of genes involved in different steps of the krill moulting cycle, in the reproductive process and in sexual maturation in accordance with what was already described in previous works. Furthermore, the new transcriptome highlighted the presence of differentially expressed genes previously unknown, playing important roles in cuticle development as well as in energy storage during the krill life cycle. The discovery of new opsin sequences, specifically rhabdomeric opsins, one onychopsin, and one non-visual arthropsin, expands our knowledge of the krill opsin repertoire. We have collected all these results into the KrillDB^2^ database, a resource combining the latest annotation of the krill transcriptome with a series of analyses targeting genes relevant to krill physiology. KrillDB^2^ provides in a single resource a comprehensive catalog of krill genes; an atlas of their expression profiles over all RNA-seq datasets publicly available; a study of differential expression across multiple conditions. Finally, it provides initial indications about the expression of microRNA precursors, whose contribution to krill physiology has never been reported before.

## Introduction

Antarctic krill *Euphausia superba* represents a widely distributed crustacean of the Southern Ocean and one of the world’s most abundant species, with a total biomass between 100 and 500 million tonnes^1^. Due to its crucial ecological role in the Antarctic ecosystem, where it represents a link between apex predators and primary producers, several studies have been carried out over the years to characterize krill distribution^2–4^, population dynamics and structuring^5, 6^ and above all to understand its complex genetics^5, 7–9^. A sizable fraction of these studies focused on the DNA, specifically on mtDNA variation; however, the information available about krill genetics remains relatively modest. The difficulty in progressing this kind of study mainly depends on the considerable large krill genome size^10^, which is more than 15 times larger than the human genome. This aspect vastly complicates DNA sequencing, which is the reason why in recent years—together with the advances in high‐throughput RNA-sequencing techniques—different krill transcriptome resources have been developed^11–16^. However, it was with the KrillDB project^17^ that a detailed and advanced genetic resource was produced and made available to the community as an organized database. KrillDB is a web-based graphical interface with annotation results coming from the de novo reconstruction of the krill transcriptome, deriving from the assembly of more than 360 million Illumina sequence reads. In this study we significantly expanded the amount of input sequences, adding 45 new samples to those used in the previous work (see Table S3: Supplementary Material), for a total of more than 4 billion RNA-seq reads. We improved the transcriptome reconstruction strategy by merging multiple independent de novo assemblies into a unique reference through the use of filters and optimization procedures.

In addition, we updated KrillDB, now renamed KrillDB^2^ (available at https://krilldb2.bio.unipd.it/). We focused on two aspects: the improvement of the quality and breadth of the krill transcriptome sequences previously reconstructed, thanks to the addition of an unprecedented amount of RNA-sequencing data; and, correspondingly, an increase in the amount of information associated with each transcript. Each transcript annotation has been extended to include its splicing structure, the predictions of orthologs, its level of abundance in different sample groups, and finally the putative secondary structure in the case of microRNA precursors.

The new krill transcriptome increased our capability to identify transcriptional phenotypes previously undetected: for instance, we recovered a greater number of differentially expressed genes involved in cuticle development and in reproduction. The analyses performed also represent a crucial step forward in the characterization of *E. superba* opsins, providing a better snapshot of the complex mechanisms underlying the krill capability to adapt to extreme diel vertical migrations and seasonal changes in light availability.

## Results

To create and annotate a de novo transcriptome assembly for Antarctic krill a preliminary investigation focusing on the efficiency and quality of already existing strategies for de novo transcriptome assembly of non-model organisms was performed. In a second step, we focused on identifying and applying the best transcriptome assembly strategy to finally explore the gene expression levels across different developmental stages and krill responses to different environmental conditions. At first, separate transcriptome reconstructions using different assembly programs were carried out. A combination of two filtering steps was applied to these results to discard artifacts and improve the assembly quality. Reconstructed transcripts across all assemblers were joined, producing a set of non-redundant representative transcripts. We obtained these results by applying the EvidentialGene pipeline (version 4), which was specifically designed to combine different reconstructions and to eliminate redundant sequences. Finally, we applied another filter to identify redundant or mis-assembled sequences still appearing in the transcriptome.

### Transcriptome quality

**Figure 1:**
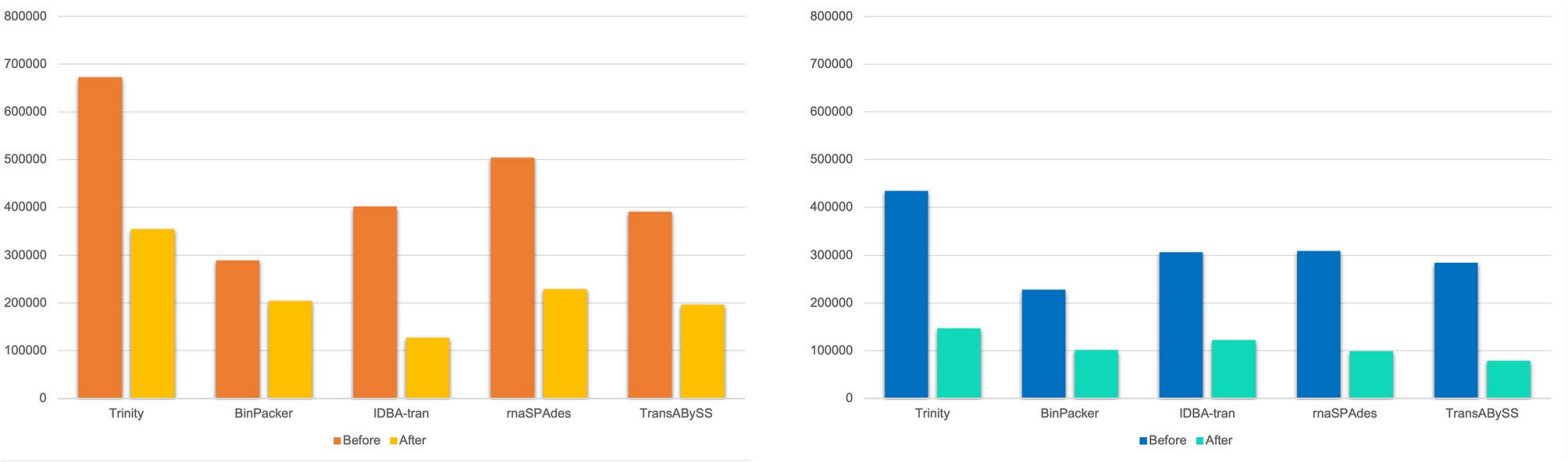
Transcriptome quality assessment results. Results of the first assembly filtering in terms of total number of transcripts.

We checked the quality of our reconstructed transcriptome step by step, starting from the independent de novo assemblies, then evaluating the potential of merging all assemblies into a unique meta-assembly, and finally filtering the transcriptome for redundancy. All these results are summarized in Fig. 1, Tables 1 and 2. The result of our reconstruction strategy was evaluated using different measures: the N50 statistics highlighted an increase in transfrag lengths at each step. Recent benchmarks, such as^18^, have shown that, while reconstructing the transcriptome of a species, no single approach is uniformly superior: the quality of each result is influenced by a number of factors, both technical (*k*-mer size, strategy for duplicate resolution) and biological (genome size, presence of contaminants). In our study, we observed that, although a consistent number of sequences was removed through each step of the assembly, merging and filtering procedure, we didn’t encounter any decline in the quality described by the basic statistics of the reconstructed transcripts (Table 1).

**Table 1:** Quality measures computed at each assembly step, from the independent de novo assembly algorithms (a), after the first filtering process (b) and finally comparing the quality of the EvidentialGene meta-assembly and the final krill transcriptome after the redundancy filter (c).

	Trinity	BinPacker	rnaSPAdes	IDBA-tran	TransABySS
**(a)**					
# Transcripts	671.837	288.476	503.293	400.75	389.351
%GC	35.56	34.66	35.39	34.77	35.07
Median contig length	368	938	347	336	332
N50	1.14	2.213	1.533	553	730
# Bases	470,615,830	413,787,317	392,082,747	198,618,480	218,288,366
**(b)**					
# Transcripts	353.34	203.274	228.038	125.886	195.764
%GC	35.77	34.80	35.61	35.18	35.17
Median contig length	452	1074	762	352	426
N50	1.455	2.317	1.958	670	1.1
# Bases	301,600,820	321,478,538	280,807,245	69,975,046	144,053,299

	EvidentialGene	KrillDB^2^			
**(c)**					
# Transcripts	274.84	151.585			
%GC	36.18	36.34			
Median contig length	756	1156			
N50	2.164	2.761			
# Bases	360,989,701	264,149,525

**Table 2:** BUSCO assessment results on independent de novo assemblies from RNA-seq stranded library.

	Complete (%)	Fragmented (%)	Missing (%)
**(a)**			
Trinity	94.4	1.3	4.3
BinPacker	94.1	0.9	5
IDBA-tran	77.9	11.5	10.6
Trans-AbySS	93.4	1.6	5
rnaSPAdes	94.6	0.8	4.6
**(b)**			
Trinity	93.3	1.7	5
BinPacker	92.9	1.7	5.4
IDBA-tran	80.9	9.0	10.1
Trans-AbySS	92.1	1.7	6.2
rnaSPAdes	93.1	1.5	5.4
**(c)**			
EvidentialGene	92.3	0.6	4.1
KrillDB^2^	93.2	0.6	6.2

(a), RNA-seq unstranded library (b) and on EvidentialGene transcriptome compared to krill transcriptome after last filter (c): the EvidentialGene transcriptome was characterized by 95.3% Complete sequences, 0.6% Fragmented and 4.1% Missing sequences. The same analysis on the final krill transcriptome reconstruction produced 93.5% Complete transcripts, 0.7% Fragmented and 5.8% Missing sequences.

We then explored the completeness of the krill transcriptome according to conserved ortholog content using BUSCO (version 4.0.5) comparing our sequences to all the expected single-copy orthologs from the Arthropoda phylum. The results of the BUSCO analyses performed on each independent de novo assembly, on the EvidentialGene reconstruction and the final transcriptome are reported in Table 2. This analysis confirms that our strategy for controlling redundancy did not affect transcriptome completeness: indeed, the fraction of complete single-copy essential genes dropped by 1.8% only, while 123,376 redundant transfrags were discarded.

We finally compared our quality assessment results with those from previously released krill transcriptomes (Table 3). Our latest assembly significantly improves all the metrics we have discussed above. While this evidence suggests that our assembly is reasonably close to providing a complete representation of the krill transcriptome, it is more difficult to gauge the amount of redundancy it contains. Specifically, it remains difficult to distinguish between splice variants of a gene and possible paralogous copies. We believe that only the availability of a genome draft will make it possible to reliably discriminate between these two signals.

**Table 3:** Quality statistics of the previously released krill transcriptomes compared to the newly assembled KrillDB^2^. GenBank accession GFCS00000000.1 refers to the SuperbaSe krill transcriptome reference^19^.

	GFCS00000000.1	KrillDB	KrillDB^2^
#Total Transcript	484.08	133.965	151.464
Median contig length	439	683	1.155
N50	1.071	1.294	2.759
BUSCO—complete	827 (81.6%)	536 (52.9%)	947 (93.5%)

### Functional classification

The assembled fragments were aligned against known protein and nucleotide databases to understand whether they could be linked to specific functions or processes described in other species. The functional annotation analyses showed that 63,903 contigs (42% of the total krill transcriptome) matched at least one protein from the NCBI NR (non-redundant) collection for a total of 98,316 unique proteins, while 62,518 transfrags found homology with a UniProtKB/TREMBL protein sequences (41% of the total), matching a total of 96,005 unique proteins. Furthermore, 22,024 krill transcripts (15% of the total) had significant matches with sequences in the NCBI NT nucleotide database. To classify transcripts by putative function, we performed a GO assignment. Specifically, 2833 GO terms (corresponding to 13,064 genes) were assigned: 1224 of those (corresponding to 11,575 genes) represented molecular functions; 1193 terms (corresponding to 6990 genes) were linked to biological processes; 416 terms (corresponding to 4301 genes) represented cellular components.

### A case study on the discovery of opsin genes

**Figure 2:**
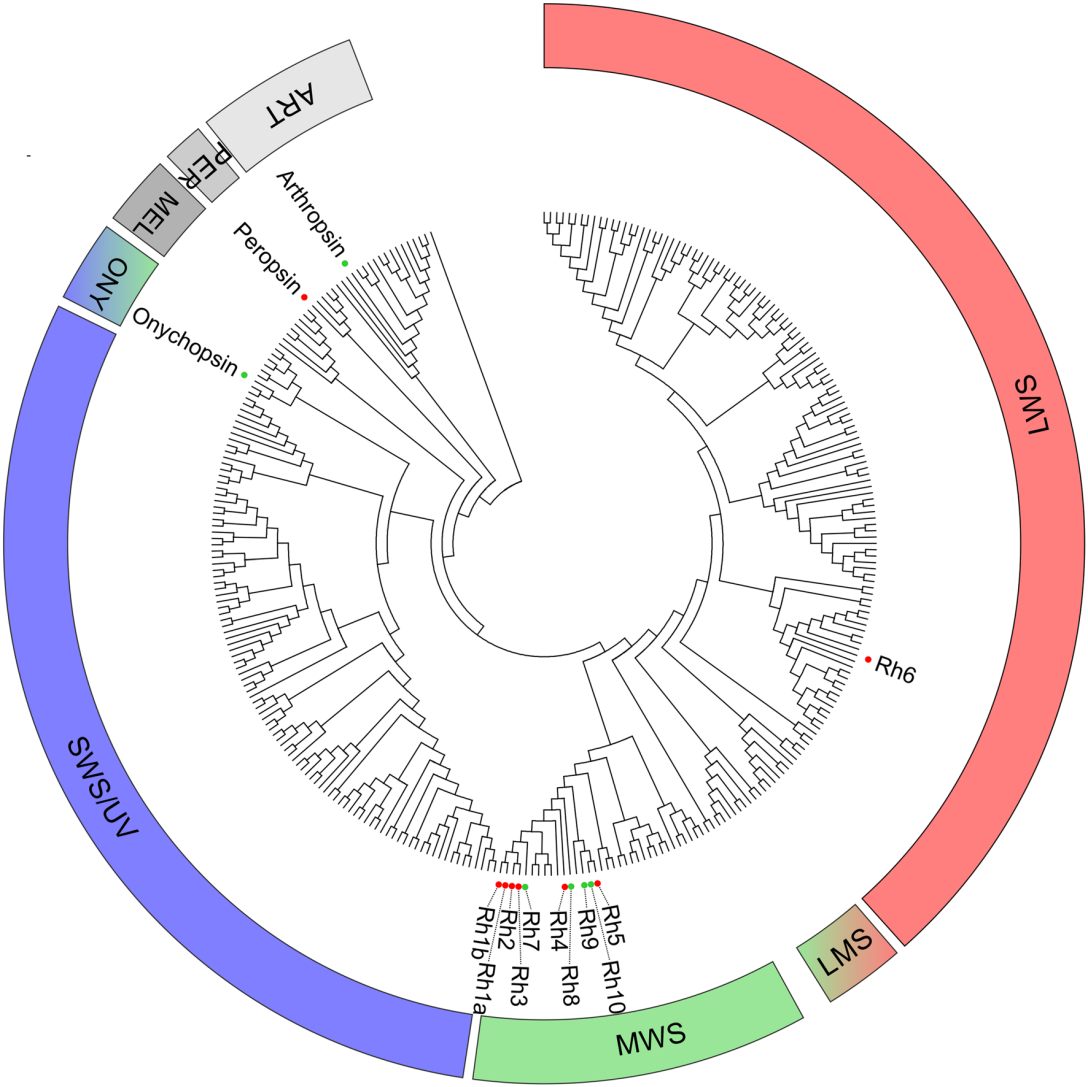
Phylogenetic relationships of *Euphausia superba* opsins shown as circular cladogram. Colored dots indicate krill opsins: red, previously cloned opsins; green, novel identified opsins. The spectral sensitivities of rhabdomeric opsin clades were inferred from the curated invertebrate-only opsin dataset proposed by DeLeo & Bracken‐Grissom, 2020. Represented opsin classes: LWS, long-wavelenght-sensitive; LSM, long/middle-wavelenght-sensitive; MWS, middle-wavelenght-sensitive; SWS/UV, short/UV-wavelenght-sensitive; ONY, onychopsins; MEL, melanopsins; PER, peropsin; ART, arthropsin. Rectangular phylogram is reported in Fig. S1 (Supplementary Material).

To evaluate the gene discovery potential of the new assembly, we searched the transcriptome for novel members of the opsin family. Opsins are a group of light sensitive G protein-coupled receptors with seven transmembrane domains. Fourteen genes were annotated as putative opsins, and the conserved domains analysis revealed that all of them possess the distinctive 7 α-helix transmembrane domain structure. The eight previously cloned opsins^20^ were all represented in KrillDB^2^ (sequence identity > 90%; Table S1 Supplementary Material). The other six genes we identified can therefore be considered new putative opsins. Among those, we found four putative rhabdomeric opsins: *Es*Rh7 and *Es*Rh8, with 70% and 59% of amino acid identity to *Es*Rh1a and *Es*Rh4, respectively; *Es*Rh9 and *Es*Rh10 showing high sequence identity (87% and 74%, respectively) to *Es*Rh5. Furthermore, we identified two putative ancestral opsins: a non-visual arthropsin (*Es*Arthropsin), and an onychopsin (*Es*Onychopsin) with 70% and 49% of sequence identity with crustacean and onychophoran orthologous, respectively. Phylogenetic analysis (Fig. 2) suggested that *Es*Rh7-10 are middle-wavelength-sensitive (MWS) rhabdomeric opsins, and further confirmed *Es*Arthropsin and *Es*Onychopsin annotation.

### Differential expression

The availability of a new assembly of the krill transcriptome, reconstructed by collecting the largest amount of experimental data available thus far, suggested the possibility of performing a more detailed investigation of differential expression patterns. Therefore, we decided to reanalyze the dataset from Höring et al.^21^ to assess the possibility of identifying differentially expressed genes that were not detected in the original study due to the use of an older reference transcriptome^15^.

Our design matrix for the model included all the independent factors (*season*, *area* and *sex*) and, in addition, the interaction between *area* and *season*, *sex* and *area*, *sex* and *season.*

In total 1741 genes were differentially expressed (DEG) among experimental conditions. They correspond to around 2% of the total reconstructed genes. In the previous work by Höring^21^, the same samples were quantified against 58,581 contigs^15^ producing 1654 DEGs. Table 4 summarizes the list of performed contrasts, each one with the number of differentially expressed up and down regulated genes.

**Table 4:** List of contrasts computed with total number of differentially expressed genes and numbers of up- and downregulated genes.

Reference condition	Alternative condition	Sample group	# Total	# Upregulated	# Downregulated
Summer	Winter	Group 2	1195	1078	117
Male	Femae	Group 2	14	7	7
Male/summer	Female/Winter	Group 2	12	6	6
South Georgia	Lazarev Sea	Group 2	79	26	53
South Georgia	Bransfield Strait-South Orkney	Group 2	28	6	22
Lazarev sea	Bransfield Strait-South Orkney	Group 2	17	13	4
South Georgia/male	Bransfield Strait-South Orkney/Female	Group 2	10	6	4
South Georgia/male	Lazarev Sea/Male	Group 2	19	8	11
South Georgia/summer	Bransfield Strait-South Orkney/Winter	Group 2	75	66	9
Lazarev Sea/summer	Bransfield Strait-South Orkney/Winter	Group 2	359	173	186
South Georgia/summer	Lazarev Sea/Summer	Group 2	188	150	38
Lazarev Sea/male	Bransfield Strait-South Orkney/Female	Group 2	20	10	10

**Figure 3:**
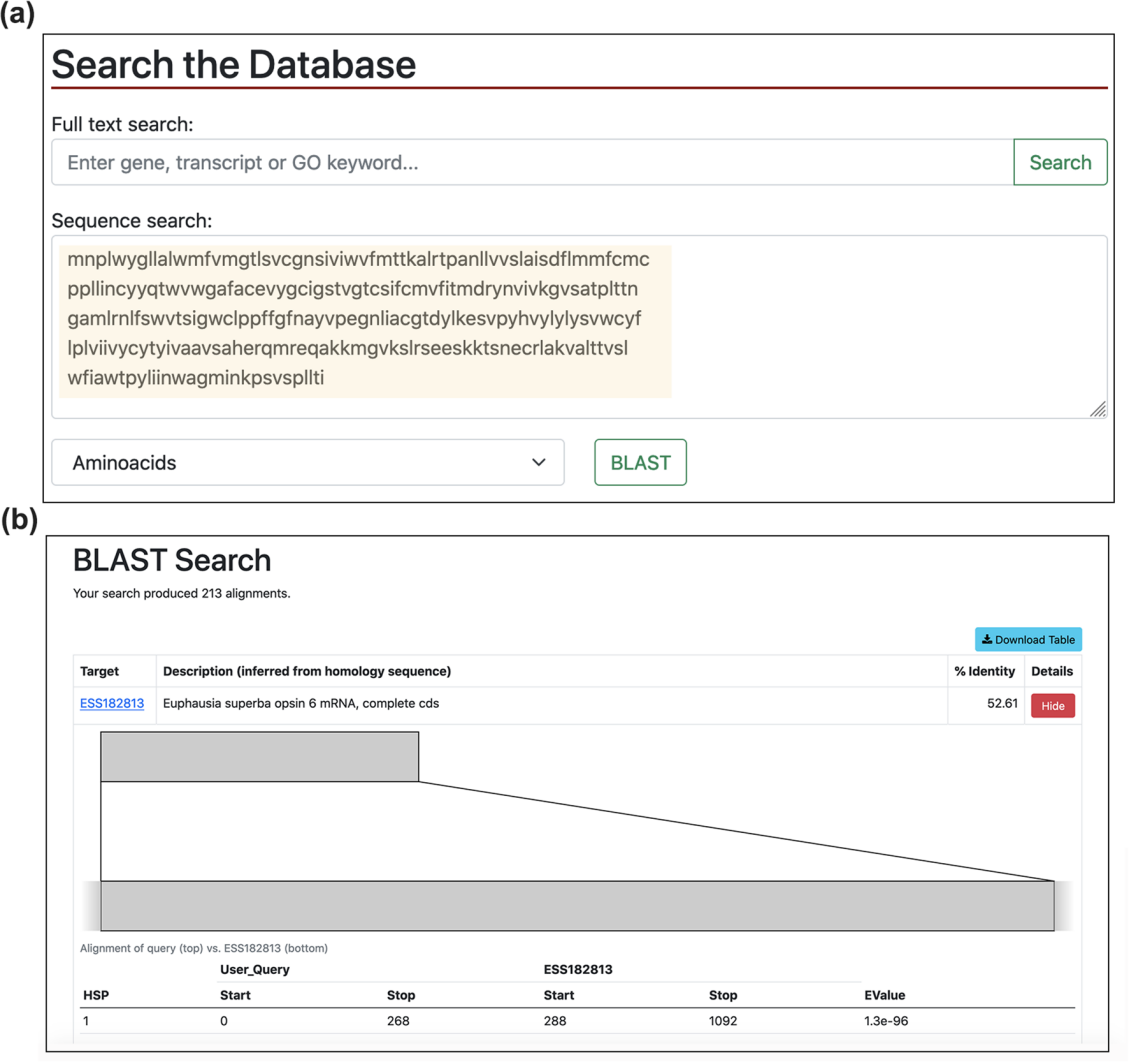
Blast search section. The new search box for sequence searches (**a**) with an example of a BLAST search (highlighted in yellow) and the corresponding results (**b**). By clicking on each target identifier, the user will be redirected to that specific transcript page, where new sections have been added, as shown in Fig. 6.

1195 DEGs were identified in the comparison between summer and winter specimens: 1078 were up-regulated and 117 down-regulated. In addition, 396 of such DEGs had some form of functional annotation. In general, these results are in accordance with the discussion by Höring^21^, which found that seasonal differences are predominant compared to regional ones. A summary of the DEGs is listed in Table 5. Complete tables of differentially expressed genes are downloadable on KrillDB^2^ (Fig. 3c; https://krilldb2.bio.unipd.it/, Section “Differentially Expressed Genes (DEGs)”).

**Table 5:** List of biologically relevant DEGs identified, starting from those already described by Höring et al.^35^.

Process	Gene	KrillDB^2^ Gene
Development of cuticle (moult cycle)	Peritrophin	ESG063925
	Chitooligosaccharidolytic beta-N- acetylglucosaminidase	ESG040750
	Carbohydrate sulfotransferase 11	ESG043538
	**Trypsin like**	**ESG046724**
	**Chitinase 1**	**ESG041912**
	**Chitinase 3**	**ESG043598**
	**Chitinase 4**	**ESG040248**
	**Endochitinase-like**	**ESG041048**
	**Glycosyltransferase 8 domain-containing protein 1-like**	**ESG047683**
	**Collagen aplha-1**	**ESG039607**
	**Glutamine-fructose-6-phosphate aminotransferase**	**ESG040051**
	**Pupal cuticle protein 20-like**	**ESG045660**
	**Early cuticle protein 3**	**ESG054542**
	**Endocuticle**	**ESG037580**
	**Crustin 1**	**ESG059398**
Immune response	Laccase	ESG048485
	Leucine rich repeat only protein 2	ESG048485
Embryogenesis	Blastula protease 10	ESG045350
Development and reproduction	Aldehyde dehydrogenase family 8	ESG043319
	Retinoid-inducible serine carboxypeptidase	ESG040940
	Dehydrogenase/reductase SDR family member 11	ESG048936
Reproduction	Vitellogenin	ESG035720
	Hematopoietic prostaglandin D synthase	ESG056241
	Carboxylic ester hydrolase	ESG040590
	Adiponectin receptor protein	ESG049090
	Type I iodothyronine deiodinase	ESG061750
	**Ovochymase 1**	**ESG044749**
	**Ovochymase 2**	**ESG052923**
	**Serine/threonine-protein phosphatase PP1-gamma catalytic subunit**	**ESG045461**
	**Doublesex and mab-3 related transcription factor 1**	**ESG045173**
Metalloendopeptidase activity	Neprilysin 1	ESG037511
Steroid metabolism	Inactive hydroxysteroid dehydrogenase-like protein 1	ESG050201
	Short-chain dehydrogenase/ reductase family 42E member 1	ESG041089
Lipid metabolism	Epoxide hydrolase	ESG048309
	Enoyl-CoA isomerase	ESG051749
	Long-chain-fatty-acid–CoA ligase	ESG040433
Glucose metabolic process	**Furin-1 precursor**	**ESG037914**
Cell cycle	**Histone-lysine M-methyltransferase MLL5**	**ESG035391**
Circadian clock	**Euphausia superba cry gene for cryptochrome, exons 1–7**	**ESG035391**
	**Vrille**	**ESG040113**
Photoreception	**Opsin 5**	**ESG047639**

Genes that were already found to be differentially expressed in the work by Höring are reported in black, while newly DEGs identified by our analysis are reported in Bold.

### Summer versus winter

We selected a series of genes among seasonal DEGs according to what has been already described in the literature. Höring et al.^21^ previously identified and described 35 relevant DEGs involved in seasonal physiology and behavior: we recovered the same gene signature in our analysis by comparing summer to winter samples. The majority of these DEGs appear to be involved in the development of cuticles (*chitin synthase*, *carbohydrate sulfotransferase 11*), lipid metabolism (*fatty acid synthase 2*, *enoyl-CoA ligase*), reproduction (*vitellogenin*, *hematopoietic prostaglandin D synthase*), metabolism of different hormones (*type 1 iodothyronine deiodinase*) and in the circadian clock (*cryptochrome*). Our results also include DEGs that were involved in the moult cycle of krill in other studies^16^. Specifically, we identified a larger group of genes involved in the different stages of the cuticle developmental process (*peritrophin-A domain*, *calcified cuticle protein*, *glycosyltransferase 8-domain containing protein 1*, *collagen alpha 1*, *glutamine-fructose 6 phosphate*), including proteins such as *cuticle protein-3,6,19.8, early cuticle protein, pupal cuticle protein, endocuticle structural glycoprotein, chitinase-3* and *chitinase-4*, the latter representing a group of chitinase which have been shown to be expressed predominantly in gut tissue during larval and/or adult stages in other arthropods and are proposed to be involved in the digestion of chitin-containing substrates^22^. Finally, in addition to *trypsin* and *crustin 4* (immune-related gene, essential in early pre-moult stage when krill still have a soft cuticle to protect them from pathogen attack, as seen by Seear et al.^16^), we also identified *crustin-1,2,3,5* and *7*. All the reported genes were up-regulated in summer, the period in which growth takes place and krill moult regularly.

Cuticle development genes were also identified as differentially expressed in the analysis of the interaction of multiple factors, between male samples coming from South Georgia and female specimens coming from the area of Bransfield Strait-South Orkney (considered as a unique area since they are placed at similar latitudes). Strikingly, we also identified a *pro-resilin* gene, whose role in many insects consists in providing efficient energy storage, being up-regulated in South Georgia male specimens.

### Interaction effects

A number of relevant DEGs were found among specific regional and seasonal factors interactions. For instance, by comparing krill samples coming from South Georgia in summer and individuals sampled in Bransfield Strait-South Orkney in winter, we found genes up-regulated in summer in South Georgia related to reproductive activities, such as *doublesex* and *mab-3 related transcription factor*. The latter is a transcription factor crucial for sex determination and sexual differentiation, which was already described in other arthropods^23^. Since no differentially expressed gene related to reproduction was found by Höring et al.^21^ in the same comparisons, this suggests that the new krill transcriptome improves the power to identify new expression patterns and characterize the krill samples.

Finally, the comparison between male individuals from the Lazarev Sea and female specimens from the Bransfield Strait-South Orkney showed additional DEGs involved in reproduction, such as *ovochymase 2*, usually highly expressed in female adults or eggs, *serine protease* and a *trypsin-like gene*. In particular, *trypsin-like genes* are usually thought to be digestive serine proteases, but previous works suggested that they can play other roles^24^; many trypsins show female or male-specific expression patterns and have been found exclusively expressed in males, as in our analysis, suggesting that they play a role in the reproductive processes.

The simultaneous presence of differentially expressed genes involved in different steps of the krill moulting cycle, in the reproductive process and in sexual maturation that appear to be differentially expressed in the same comparisons is in accordance with what was already observed in krill^25^ and other krill species^26^. In particular, there is evidence of a strong relation between the krill moulting process and its growth and sexual maturation during the year, which supports and confirms the reliability of our results in terms of genes involved in such krill life cycle steps.

### Identification of microRNA Precursors

Although microRNAs play a key role in the regulation of gene expression and in many important biological processes, such as development or cell differentiation, there is still no information about microRNAs in krill species.

Here we performed an investigation to test whether the new transcriptome could also include sequences with a significant homology to known mature microRNAs.

In total we identified 261 krill transcripts whose sequences are highly similar to 644 known microRNAs from other species. 306 sequences were linked to at least one GO term, matching 54 krill transcripts (Table S2, Supplementary Material). Among them, we identified 5 putative microRNAs involved with changes in cellular metabolism (age-dependent general metabolic decline—GO:0001321, GO:0001323), as well as changes in the state or activity of cells (age-dependent response to oxidative stress—GO:0001306, GO:0001322, GO:0001324), 35 microRNAs involved in interleukin activity and production. We found 26 putative microRNAs likely involved in *ecdysteroidogenesis* (specifically GO:0042768), a process resulting in the production of ecdysteroids, moulting and sex hormones found in many arthropods. In addition, we found a microRNA involved in fused antrum stage (GO:0048165) which appears to be related in other species to oogenesis. We also identified 27 microRNAs related to *rhombomere* morphogenesis, formation and development (GO:0021661, GO:0021663, GO:0021570). These functions have been linked to the development of portions of the central nervous system in vertebrates, which share the same structure of those found in arthropod brains. Lastly, 26 krill sequences showed high similarity with 2 mature microRNA related to the formation of tectum (GO:0043676), which represents in arthropods and, specifically, crustaceans, the part of the brain acting as visual center.

### KrillDB^2^ web Interface

**Figure 4:**
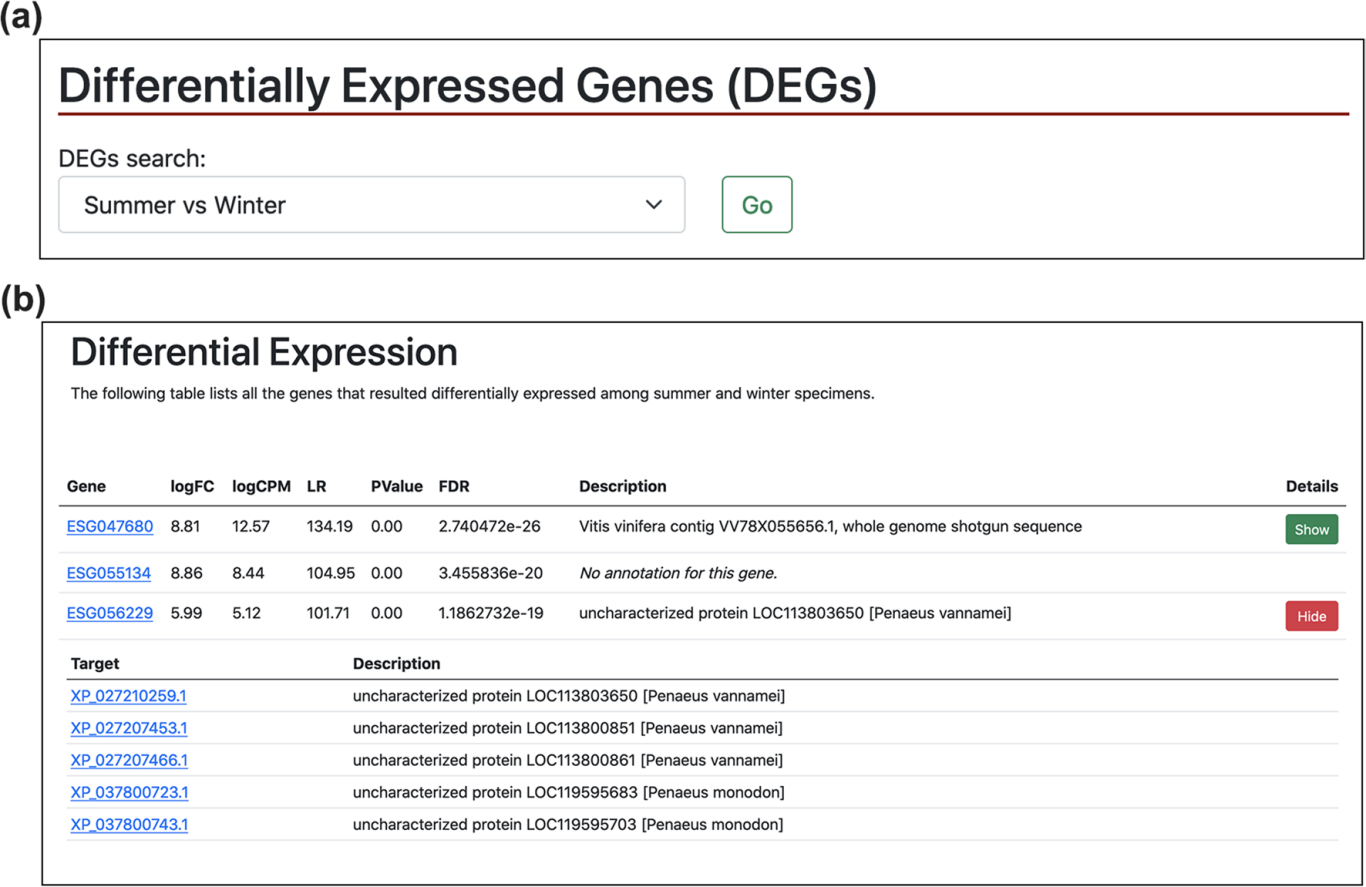
Differential Expression section. The new section collecting all differentially expressed genes tables (**a**) with an example of the corresponding result for a selected contrast (**b**).

**Figure 5:**
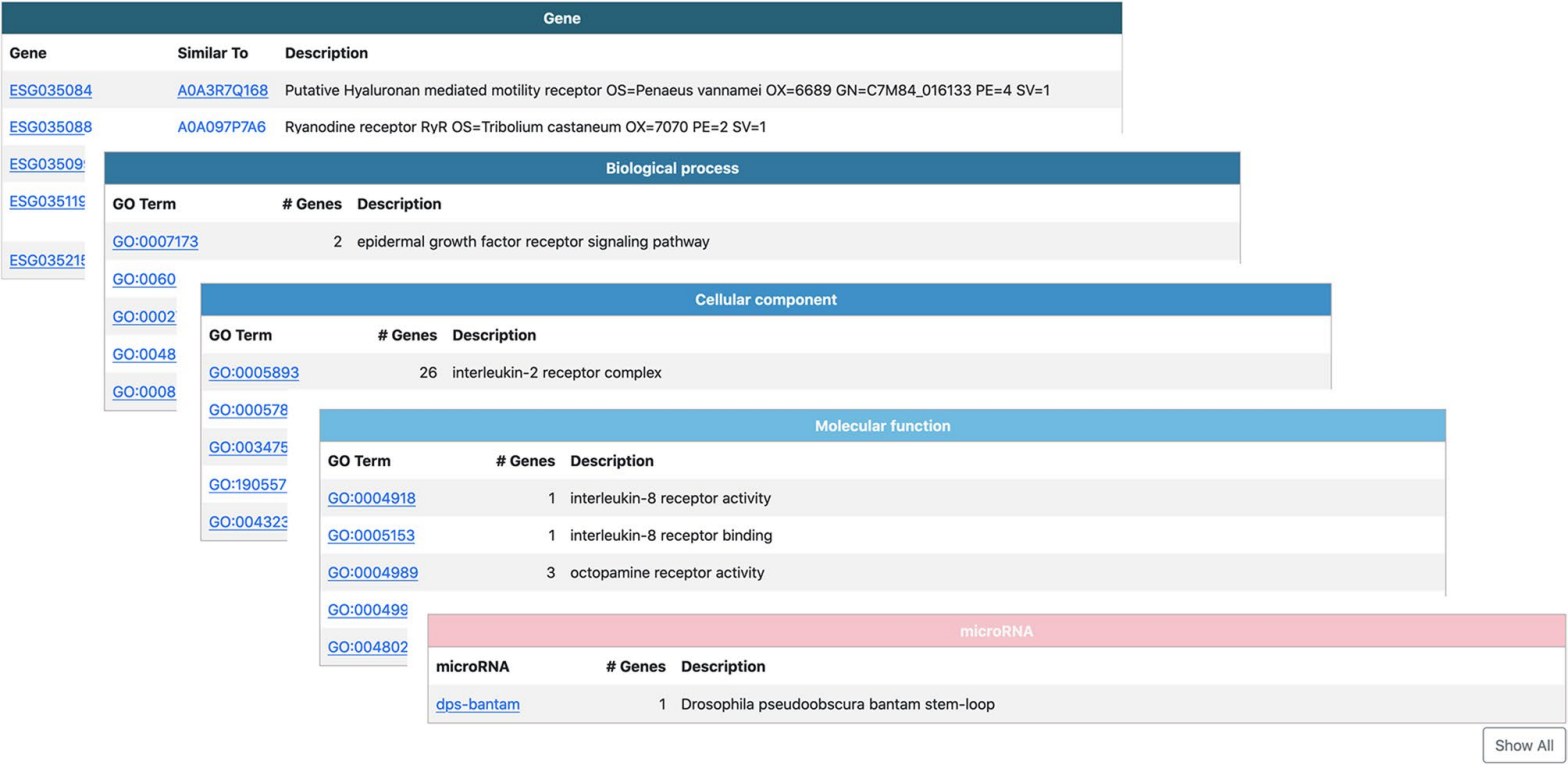
New search engine of KrillDB^2^. Example of the results of a full-text search on KrillDB^2^.

**Figure 6:**
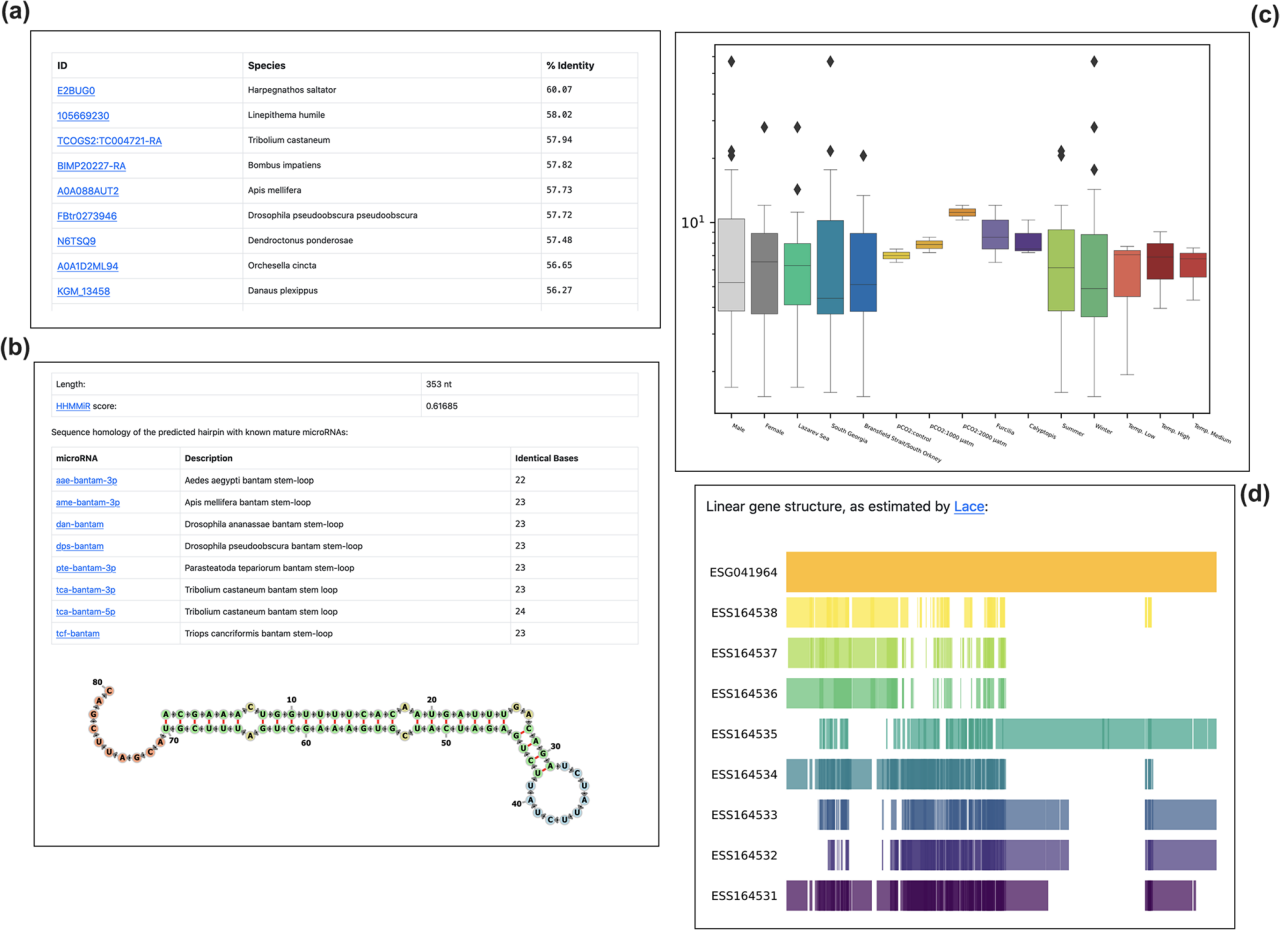
Additional sections in gene and transcript pages. The new sections in the gene-centric page show a table listing the orthologous sequences with their belonging species and the identity score (**a**), a visualization of the gene structure as estimated by Lace software (**d**) and a boxplot coming from Expression Atlas analyses (**c**). Both Orthology and Expression sections are integrated also in the transcript-centric page. When a transcript is annotated as a putative microRNA, a “Predicted Hairpin” section displays a visualization of the hairpin predicted secondary structure and tables showing the alignment length, the HHMMiR score and the list of mature microRNAs matching (**b**).

The KrillDB website has been redesigned to include the new version of the transcriptome assembly. Figures 3, 4, 5 and 6 collect images taken from the new main sections of the database. The integrated full-text search engine allows the user to search for a transcript ID, gene ID, GO term, a microRNA ID or any other free-form query. Results of full-text searches are now organized into several separate tables, each representing a different data source or biological aspect (Fig. 5). Results of GO term searches are summarized in a table reporting the related genes with corresponding domain or microRNA match and associated description. Both gene and transcript-centric pages have been extended with two new sections: “Orthology'' and “Expression” (Fig. 6). The Orthology section summarizes the list of orthologous sequences coming from the OMA analysis, each one with the species it belongs to and the identity score.

The “Expression” section shows a barplot representing abundances estimates obtained from Salmon. An additional section, called “Gene Structure” (Fig. 6), was added to the gene page on the basis of the results coming from the SuperTranscript analysis. Specifically, we modified the STViewer.py Python script (from Lace), optimizing and adapting it to our own data and database structure, in order to produce a visualization of each gene with its transcripts. Since Lace relies on the construction of a single directed splice graph and it is not able to compute it for complex clusters with more than 30 splicing variants, this section is available for a selection of genes only.

The new KrillDB^2^ release includes completely updated transcript and gene identifiers. However, the user searching for a retired ID is automatically redirected to the page describing the newest definition of the appropriate transcript or gene.

The KrillDB^2^ homepage now includes two additional sections: one is represented by the possibility to perform a BLAST search (Fig. 3). Any nucleotide or protein sequence (*query*) can be aligned against krill sequences stored in the database. Results are summarized in a table containing information about the krill transcripts (*target*) that matched with the user’s query, and the e-value corresponding to the alignment. The other new section, called “Differentially Expressed Genes”, allows the user to browse all the tables listing the genes that were found to be differentially expressed among the conditions we have described above (Fig. 4). A drop-down menu gives access to the different comparisons; DEG tables list for each gene its log fold-change, p- and FDR values as estimated by edgeR. Moreover, each gene is linked to a functional description (if available) inferred from sequence homology searches.

Information about krill transcripts showing homology with an annotated microRNA is available in the “Predicted Hairpin” (Fig. 6). It contains a summary table with details about the hairpin length and the similarity score (as estimated by HHMMiR), followed by full listing of all the corresponding mature microRNAs (including links to their miRBase page). In addition, an image displaying the predicted secondary structure of the hairpin is included (computed by the “fornac” visualization software from the ViennaRNA suite).

## Discussion

The availability of a large amount of public RNA-seq data capturing krill transcripts has allowed us to re-assemble its transcriptome and to significantly extend its annotation. We have now covered the entire developmental process of this species and included in our analysis individuals belonging to different seasons and affected by different environmental conditions. KrillDB^2^ provides the most complete source of information about the krill transcriptome and will offer a reliable starting point for the development of novel ecological studies. As shown in Fig. 1, Tables 1 and 2, the analysis of the quality of previously released krill transcriptome in comparison to the newly assembled KrillDB^2^ confirmed how the strategy applied did not produce any loss in terms of quality, although a consistent number of transcripts was removed. The quality metrics, in contrast, were improved both in terms of N50 statistics and transcriptome completeness: the fraction of complete single-copy essential genes reached 93.2%.

The differential expression analysis we have performed highlights the importance of specific processes in the complex krill life cycle and in its adaptation capability to the harsh Antarctic environment.

Identifying six novel putative opsin sequences almost doubles the eight previously cloned, demonstrating a significant improvement in the gene discovery potential of this new version of krill transcriptome. The finding of four novel MWS rhabdomeric opsins, an onychopsin, and a non-visual arthropsin further enrich the opsin repertoire of *E. superba* shedding light on a complex photoreception system able to coordinate the physiological and behavioral responses to the extreme daily (diel vertical migration) and seasonal changes in photoperiod and spectral composition. Arthropsins are rhabdomeric non-visual opsins and its clade is the sister group of the bilaterian rhabdomeric opsins^27, 28^. It was first discovered in the crustacean *Daphnia pulex* and subsequently in other arthropods, onychophoran, molluscs, annelids and flatworms^27–31^. Of relevance is the identification of an onychopsin which has been suggested to be the common ancestor of *Panarthropoda* visual opsins ^27^, and possibly sensitive to wavelength from UV to green light^32^. *Es*Onychopsin could represent the short-wavelength sensitive opsin (SWS/UV) which we have long been searching for. Indeed, the absence of a SWS/UV opsin was truly unexpected in an organism that shows daily vertical migration reaching depth beyond the 30 m, where only short wavelength light can penetrate.

Finally, KrillDB^2^ includes initial evidence about the presence of non-coding RNAs in krill, specifically sequences likely corresponding to microRNAs precursors. Although this is just a preliminary analysis, the results we have described already hint at a role of microRNAs in defining the adaptive capabilities of this species to the Antarctic environment. This represents a starting point for the study of non-coding RNAs in the Antarctic krill and in other species belonging to the same family.

## Material and methods

### Krill collection

This study aims at covering the entire developmental process of krill. Therefore, we used samples coming from different developmental stages to cover the entire *E. superba* transcriptome, from larval to adult specimens. Specifically, adults included both male and female specimens, as well as summer and winter individuals and they also came from 3 different geographical regions: Lazarev Sea, South Georgia, and Bransfield Strait/South Orkney. The entire samples collection used to produce the new transcriptomic reference and carry out all downstream analysis is listed in Table S3 (Supplementary Material).

### Transcriptome assembly strategy

#### Multiple independent de novo assemblies

**Figure 7:**
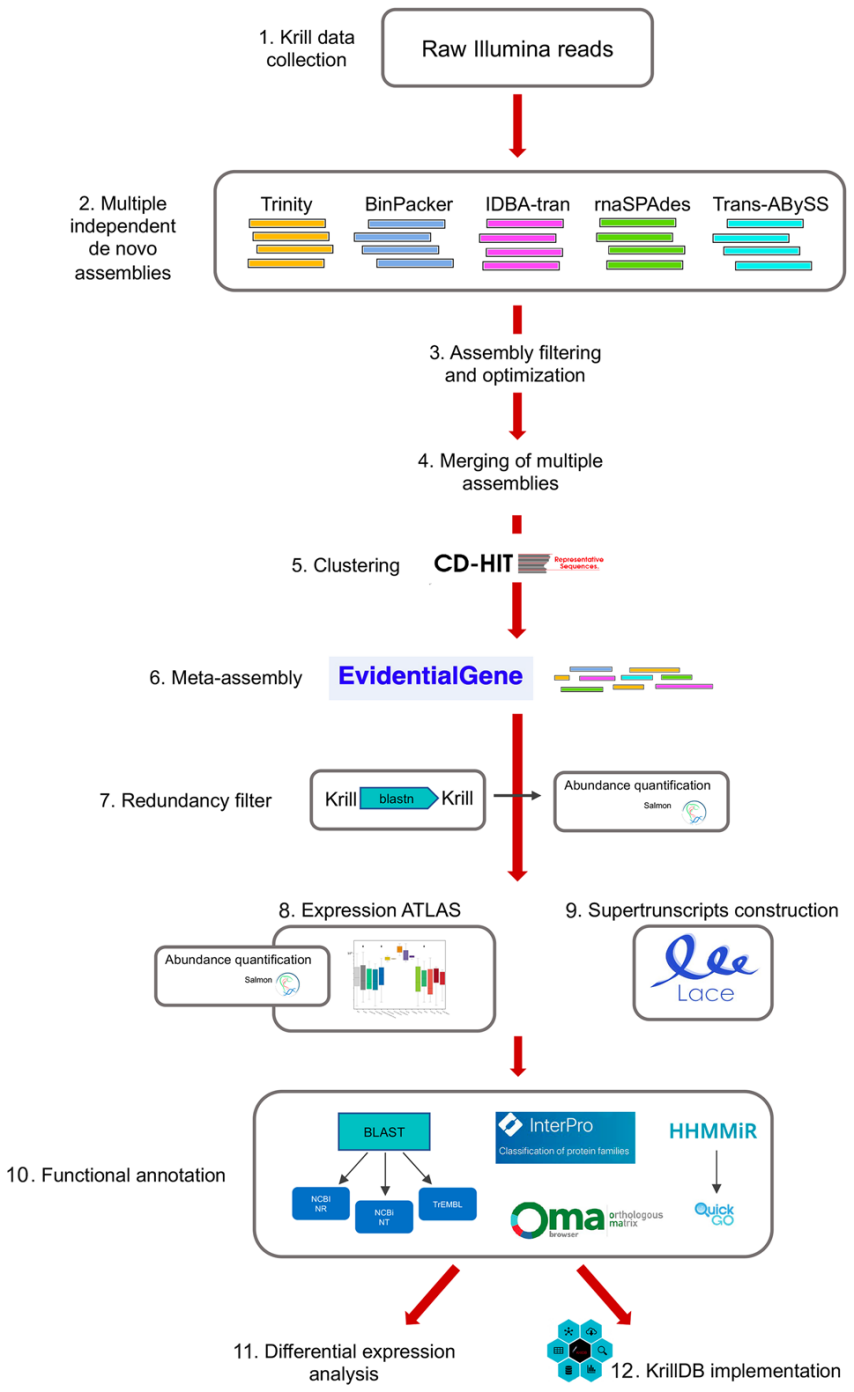
Workflow of the assembly process, annotation, database re-design and downstream analyses.

The assembly of short (Illumina) reads to reconstruct the transcriptomes of non-model organisms has been subject to a considerable amount of research. Out of the many tools developed for this task, we selected the five which are arguably the most popular in the field: Trinity (version 2.11.0)^33^, BinPacker (version 1.0)^34^, rnaSPAdes (version 3.14.1)^35^, TransABySS (version 2.0.1)^36^ and IDBA-tran (version 1.1.3)^37^. We summarized all the steps of the assembly reconstruction strategy, annotation process and downstream analyses in Fig. 7.

At first, we performed a separate transcriptome reconstruction with each of the tools listed above. Then, we evaluated their respective advantages through a series of independent measures, such as: the total number of transcripts; %GC content; the average fragment length; the total number of bases; the N50 value; and finally, the results of the BUSCO analysis, which provides a measure of transcriptome completeness based on evolutionarily informed expectations of gene content from near-universal single-copy orthologs^38^.

### Assembly filtering and optimization

The raw sequencing data we used for the assemblies was obtained from different experiments and included both stranded (Table S3 Group 2) and unstranded libraries (Table S3 Group 1). As mixing these two types of libraries in a single assembly is not well supported, we decided to run each software twice: we thus generated a total of ten different de novo assemblies.

We used Trimmomatic^39^ to remove adapter sequences and other artifacts from raw Illumina sequences. The quality of trimmed reads was checked with the program FastQC^40^ (version 0.11.9). De novo transcriptome assembly was performed using specific parameters depending on the library type (the actual commands used are listed in Table S4, Supplementary Material).

Once assembled, a combination of two filtering steps was then applied to the newly reconstructed transcriptomes to discard artifacts and improve the assembly quality.

First, we estimated the abundances of all the transcripts reconstructed by each assembler using the Salmon software^41^ (version 1.4.0). Specifically, we used the following parameters were used: samples coming from unstranded library (Table S3, Group 1) were aligned using the options “-l ISR -1—validateMappings”; samples coming from stranded library (Table S3, Group 2) were aligned using the options “-l IU—validateMappings”. Samples were grouped according to the main experimental conditions: (1) sex, with female and male levels; (2) geographical area, covering Bransfield Strait, South Georgia, South Orkney and Lazarev Sea; and (3) season, with summer and winter levels. Abundance estimates were imported in the R statistical environment using the *tximport* package^42^ and we implemented a filter to keep only those transcripts showing an expression level of at least 1 transcript per million (TPM) within each of the three experimental conditions.

In a second step, we considered the results of all assemblers jointly, and we ran the “cd-hit-est” program (version 4.8.1)^43^, with parameters set as follows: -c 0.95-M 100000-T 22. This analysis was performed in order to cluster similar sequences and to produce a set of non-redundant representative transcripts. Specifically, we collapsed all sequences sharing 95% or more of their content, thus reducing the number of transcripts from 1,650,404 to 551,110.

### Meta-assembly

The procedure described above was designed to identify near-duplicate sequences deriving from different software, but likely corresponding to the same biological transcript. As a further refinement, we were also interested in grouping resulting transcripts into units corresponding to genes. To this end, we relied on the EvidentialGene pipeline (version 4)^44, 45^. We applied the “tr2aacds” tool which clusters transcripts and classifies them to identify the most likely coding sequence representing each gene. EvidentialGene clustering was therefore applied using the following parameters: -NCPU = 22 -MAXMEM = 100000 -logfile -tidyup -species = Euphausia_superba. The software subdivides sequences into different categories, including primary transcript with alternates (main), primary without alternates (noclass), alternates with high and medium alignment to primary (althi1, althi, altmid) and partial (part) incomplete transcripts. A “coding potential” flag is also added, separating coding from non-coding sequences (see “KrillDB2 Web Interface” section). The meta-assembly thus obtained consisted in 274,840 putative transcripts, subdivided into 173,549 genes.

As these figures remained high, we performed another round of analyses to identify redundant or mis-assembled sequences still appearing in our transcriptome. Here we used a combination of BLAST searches against known protein and nucleotide databases (NR, NT, TREMBL) and information deriving from full-length, experimentally validated transcripts from a previous study^46^. Results confirmed that the newly reconstructed transcriptome fully represented krill RNAs, but the large amount of input reads, together with the number of independent de novo assemblers, likely led to an inflation in the number of alternative splicing variants being reconstructed. Moreover, transcript alignments against BUSCO genes^38^ and the *doubletime*, *cry1, shaggy* and *vrille* full-length transcripts from^46^ highlighted the fact that multiple fragments of the same gene were incorrectly assembled as separate transfrags. To remove these artifacts, first we aligned all transcript sequences in our meta-assembly against each other using the *blastn* tool. We discarded all sequences already included in a longer transcript for more than the 90% of their length. This filter helped us remove 78,731 redundant sequences (29% of transcripts, overall). Then, we ran a new abundance quantification using Salmon and we discarded all transcripts with an average abundance below 0.1 TPM.

The combination of all the filters discussed above allowed us to reduce the number of transcripts to 151,464 and, correspondingly, that of genes to 85,830. Our approach discarded redundant genes, while retaining alternative transcripts with a sufficient level of uniqueness in their sequence. This was confirmed by the fact that although we removed almost 45% of the initially assembled transcripts, this filtering barely affected the average read mapping rate, which went from 89% (initial EvidentialGene output) to 88% (full filtering). All samples appeared to be well represented in the reference transcriptome as confirmed by the fact that the average read mapping rates from each sample group was comparable (Group 1: 89%; Group 2: 88%; Group 3: 88%; Group 4: 90%).

In order to enhance the interpretability of the transcriptome reconstruction, we also employed a SuperTranscripts analysis, on the basis of the workflow proposed by^47^. Specifically, we ran the Lace software (https://github.com/Oshlack/Lace) to reconstruct the block structure of each gene (see “KrillDB2 Web Interface” section).

### Functional annotation

Assembled fragments were aligned against the NCBI NR (non-redundant) and UniProtKB/TrEMBL protein databases, and against the NCBI NT nucleotide collection (data downloaded on 22/04/2021). We also ran InterproScan (version 5.51-85.0) to search for known functional domains and to predict protein family membership. Results with an *e-value* greater than 1e-6 for proteins (*blastx*) or 1e-9 for nucleotides (*blastn*) were discarded.

Gene orthology inference was performed using the Orthologus MAtrix (OMA) standalone package^48^ (https://omabrowser.org/standalone/) which relies on a complete catalog of orthologous genes among more than 2300 genomes covering the entire tree of life. This analysis helped us identify, based on protein sequences, those krill transcripts showing an orthology relationship with genes from other species and which sets of genes derived from a single common ancestral gene at a given taxonomic range^49^.

Finally, all krill transcripts were compared against the RNAcentral database (https://rnacentral.org/; https://doi.org/10.1093/nar/gkw1008) in order to identify any homology with the mature sequences of known microRNAs from other species.

### Expression atlas

We used the final assembly described above to re-estimate transcript abundances over a wide range of RNAseq dataset (see Table S3) including:

- Larval krill at two different stages of development exposed to different CO_2_ conditions, coming from^17^ (Table S3, Group 1)
- Adult krill (48 samples) coming from different geographical areas (Bransfield Strait, Lazarev Sea, South Georgia, South Orkney) and different seasons (summer and winter), divided into male and female specimens^21^ (Table S3, Group 2)
- Adult krill exposed to three different temperatures—Low Temperature, Mid temperature, High Temperature (Table S3, Group 3)
- Adult krill divided into male and female specimens^50^ (Table S3, Group 4)

Overall, these datasets include six experimental factors: geographical area, season, developmental stage, pCO_2_ exposure condition, sex and temperature. Newly computed transcript abundances and raw counts were imported using R (version 4.0.5) and the package *tximport* (version 1.18.0). Batch effect removal was performed using the *removeBatchEffect* function implemented in the *limma* package (version 3.46.0). The resulting count matrix of transcripts (rows) across samples (columns) was then converted to the transcripts per million (TPM) scale. Finally, results were summarized to the gene level using the *isoformToGeneExp* function (IsoformSwitchAnalyzeR version 1.12.0). The expression levels for each experimental condition are displayed in KrillDB^2^ as a boxplot, as part of the webpage for each gene or transcript (see “KrillDB2 Web Interface” section).

### Differential expression analysis

Transcript-level abundances and estimated counts were summarized at the gene-level using the package *tximport*. Resulting counts were normalized to remove unwanted variation by means of the RUVg method^51^. Specifically, we performed a preliminary between-sample normalization (EDASeq, version 2.24.0) to adjust for sequencing depth. Following the workflow outlined in the RUVseq vignette, we identified a set of negative control genes with an FDR level larger than 0.8. We applied the RUVg method to estimate k = 2 factors of unwanted variation and we included those in the design matrix for the final differential expression analysis, performed using the GLM method implemented by the edgeR software (version 3.32.1). All p-values were corrected using the Benjamini–Hochberg method.

### MicroRNAs

We also investigated the possibility that the new transcriptome included sequences corresponding to the precursors of krill microRNAs.

To this aim, we ran the HHMMiR software^52^, which combines structural and sequence information to train a Hierarchical Hidden Markov Model for the identification of microRNA genes. We also performed a *blastn* search of all our assembled transcripts against the collection of miRBase (http://www.mirbase.org/) mature sequences. Results from these two analyses were combined: we collected all transcripts with a HHMMiR score below or equal to 0.71 and an alignment to a known mature microRNA with at most two mismatches. We then used the QuickGO tool (https://www.ebi.ac.uk/QuickGO/) to identify any potential association among our putatively identified microRNA precursors and GO categories.

### Opsin phylogeny

To identify novel opsin genes in krill, we manually examined the list of transcripts that were annotated as “opsin” by our automated pipeline. Furthermore, the entire krill transcriptome was aligned against a curated opsin dataset (including 996 visual and non-visual opsins^53^) using Blast+(version 2.11.0). For genes with multiple alternative variants, we selected the longest transcript as a representative sequence. Secondary structure was assessed by the NCBI Conserved Domain Search (CDD database, May 2021). A phylogenetic tree was generated using the MUSCLE alignment tool and the Maximum Likelihood method (Dayhof substitution matrix and Nearest-Neighbor-Interchange method) as implemented in MEGA X (version 10.2.6, https://www.megasoftware.net/). New opsins were aligned against a curated invertebrate-only opsin data set^54^, the previously cloned krill opsins^20^, and the full-length onychopsin and arthropsin sequences available on the NCBI Protein database (May 2021, ncbi.nlm.nih.gov/protein). The tree was rooted using the human G protein-coupled receptor VIPR1 as an outgroup. Data S1 (Supplementary Material) includes the multi-alignments performed. Data S2 (Supplementary Material) contains all the protein sequences used to produce the tree.

### Web interface implementation

The website was developed as a Python application based on the Flask framework. Data is stored in a PostgreSQL 12.8 database (http://www.postgresql.com). The sequences of the assembled transcripts and corresponding proteins are available for download as FASTA files. Gene and transcript pages have been updated with boxplots implemented using the Seaborn Python library (version 0.11.1).

## Supplementary Information

Supplementary Information 1.

Supplementary Information 2.

Supplementary Information 3.

Supplementary Information 4.

Supplementary Information 5.

Supplementary Information 6.

## Data Availability

Data used for the krill transcriptome reconstruction and for the generation of the Expression Atlas was downloaded from the NCBI Short Read Archive, under accessions: PRJEB30084, PRJNA362526, PRJEB30084, PRJNA362526 and PRJNA640244.

## Code availability

The scripts used in this research to assemble the krill transcriptome are listed in Table S4 (Supplementary Material).
