# Supplementary material for: A thorough annotation of the krill transcriptome offers new insights for the study of physiological processes
Source: Sci Rep. 2022 Jul 6;12:11415. doi: 10.1038/s41598-022-15320-5 (PMC9259678; doi:10.1038/s41598-022-15320-5)
Supplement: Supplementary file 3 — Supplementary Information 3. [file 41598_2022_15320_MOESM3_ESM.pdf]

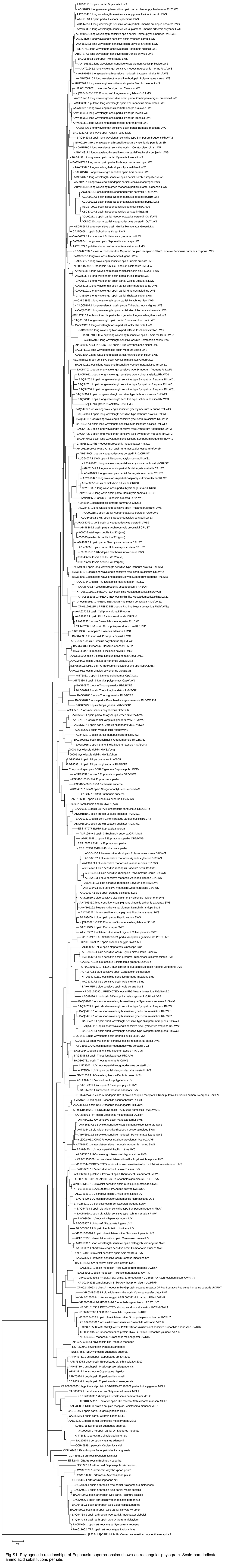

Fig S1. Phylogenetic relationships of Euphausia superba opsins shown as rectangular phylogram. Scale bars indicate amino acid substitutions per site.
