# Supplementary material for: A thorough annotation of the krill transcriptome offers new insights for the study of physiological processes
Source: Sci Rep. 2022 Jul 6;12:11415. doi: 10.1038/s41598-022-15320-5 (PMC9259678; doi:10.1038/s41598-022-15320-5)
Supplement: Supplementary file 4 — Supplementary Information 4. [file 41598_2022_15320_MOESM4_ESM.docx]

Supplementary Material

**Table S1. List of the 14 *Euphausia superba* putative opsins.**

**Table S4. Command line for each *de novo* assembly reconstruction performed.** The column “Sample Group” refers to sample annotation as reported in Table S1. The command line for IDBA-tran program was the same for both sample groups as this software does not provide any option regarding the library type.

**Data S1. Multiple sequence alignment produced using MEGA software.** Multiple sequence alignments of opsin proteins performed to produced the phylogenetic tree.

**Data S2. Protein sequences used to produce the opsins phylogenetic tree.** Curated list of opsin sequences including invertebrate-only opsin data set [[54](#bookmark=id.46r0co2)], the cloned krill opsins from Biscontin et al. (2016) [[20](#bookmark=id.3j2qqm3)], and the full-length onychopsin and arthropsin sequences available on the NCBI Protein database (May 2021, ncbi.nlm.nih.gov/protein).
